# Genetic description of a tick-borne encephalitis virus strain Sofjin with the longest history as a vaccine strain

**DOI:** 10.1186/s40064-015-1561-y

**Published:** 2015-12-09

**Authors:** M. F. Vorovitch, L. I. Kozlovskaya, L. Iu. Romanova, L. L. Chernokhaeva, A. A. Ishmukhametov, G. G. Karganova

**Affiliations:** Federal State Unitary Enterprise on Manufacture of Bacterial and Viral Preparations of Chumakov Institute of Poliomyelitis and Viral Encephalitides, Moscow, 142782 Russia; Chumakov Institute of Poliomyelitis and Viral Encephalitides, Institut Poliomyelita, Moscow, 142782 Russia

**Keywords:** TBE vaccine, Strain Sofjin, TBEV

## Abstract

Vaccines based on the strain Sofjin of the Far-Eastern tick-borne encephalitis virus (TBEV) subtype have been used for TBE prophylaxis for over 50 years in Russia and neighboring countries. On the wide territory, where all known TBEV subtypes are circulating, the cultural, purified, concentrated, inactivated TBE vaccine Moscow has been shown to be safe and efficacious in a massive immunization. In the present work, we describe the genome of the vaccine strain Sofjin. We have shown that it differs from TBEV strains previously published with the name “Sofjin”. Moreover, we have shown the stability of the virus during the vaccine manufacturing process on the molecular level.

## Background

The tick-borne encephalitis virus (TBEV) affects many people in Eurasia, causing about 10,000 cases annually. TBEV belongs to the *Flavivirus* genus. Its enveloped virion is covered by membrane-linked glycoprotein E and protein M underlying protein E. This protein-membrane cage encloses genomic RNA packed with protein C (Lindenbach et al. 2013). TBEV is divided into three subtypes: European, Siberian, and Far-Eastern (King et al. 2012), and two newly described groups of TBEV strains are different from each other and from other genotypes (Demina et al. 2010).

TBEV strain Sofjin was isolated from the brain of a patient with acute TBE in Primorskiy kray in 1937 (Smorodintseff et al. 1941), and was used for the production of the TBE vaccine in Moscow (FSUE Manufacture of Bacterial and Viral Preparations of Chumakov Institute of Poliomyelitis and Viral Encephalitides, FSUE IPVE, Russia) for over 50 years. Starting in the 1960s, the inactivated vaccine was prepared from the cultural fluid of chicken embryo fibroblasts (CEF), and, since 1982, over 33,000,000 doses of the concentrated inactivated lyophilized vaccine have been distributed.

In 1986, the genome fragment, encoding proteins C, M and E, of the TBEV strain Sofjin, was sequenced (Pletnev et al. 1986). The complete genome sequence was presented only in (Pletnev et al. 1990). Over the next 20 years, more sequences appeared, describing the nucleotide composition of the strains called Sofjin (Kovalev et al. 2012), drastically differing one sequence from one another. This fact arouses the necessity for the description of the strain Sofjin particularly used for the TBE vaccine produced in Moscow.

Currently, two preparations of the TBE vaccine Moscow from TBEV strain Sofjin, produced by FSUE IPVE, are used for preventive vaccination against TBE: a cultural, purified, concentrated, inactivated, lyophilized vaccine for the vaccination of children 3 years of age and up, and a cultural, purified, concentrated, inactivated, suspension adsorbed vaccine “Tick-E-Vac”, for children from 1 to 16 years at a dose of 0.25 ml, and for persons 16-years-old and up in a dose of 0.5 ml. Both preparations are manufactured by similar technological processes.

In 1962 the TBEV strain Sofjin was provided by M.P. Chumakov’s laboratory to the FSUE IPVE to be used as a master seed bank for the vaccine. The master seed bank was multiplied twice in 1977 and 1985 via the passage through a mouse brain of TBEV strain Sofjin, then aliquoted and stored. Nowadays, during the technological process, one aliquot from the master seed bank (from 1985) is multiplied 2–3 times in a mouse brain (working seed bank), and then multiplied in a CEF (non-concentrated bulk). Non-concentrated bulks are being inactivated with formaldehyde, concentrated via ultrafiltration, and purified through gel-filtration and protamine sulfate to obtain an inactivated vaccine lot.

We obtained complete genome sequences for a master seed bank and working seed bank viruses, and sequences of structural parts of the genomes of non-concentrated bulk viruses. Viral RNA was extracted from mice brain suspensions or CEF culture supernatants with TRIReagent LS (Sigma-Aldrich), and, after RT-PCR with overlapping sets of specific primers (sequences available upon request), M-MLV reverse transcriptase (Promega) and Taq-polymerase (Fermentas), was sequenced directly from PCR-DNA on the ABI PRISM 3730 sequencer using ABI PRISM^®^ BigDye™ Terminator v. 3.1.

The master seed bank nucleotide sequence of the genome fragment, encoding the whole polyprotein, was deposited into GenBank (ID KC806252), and the sequence was named “Sofjin-Chumakov” to differentiate it from the other strains with the same name deposited to the GenBank previously.

**Fig. 1:**
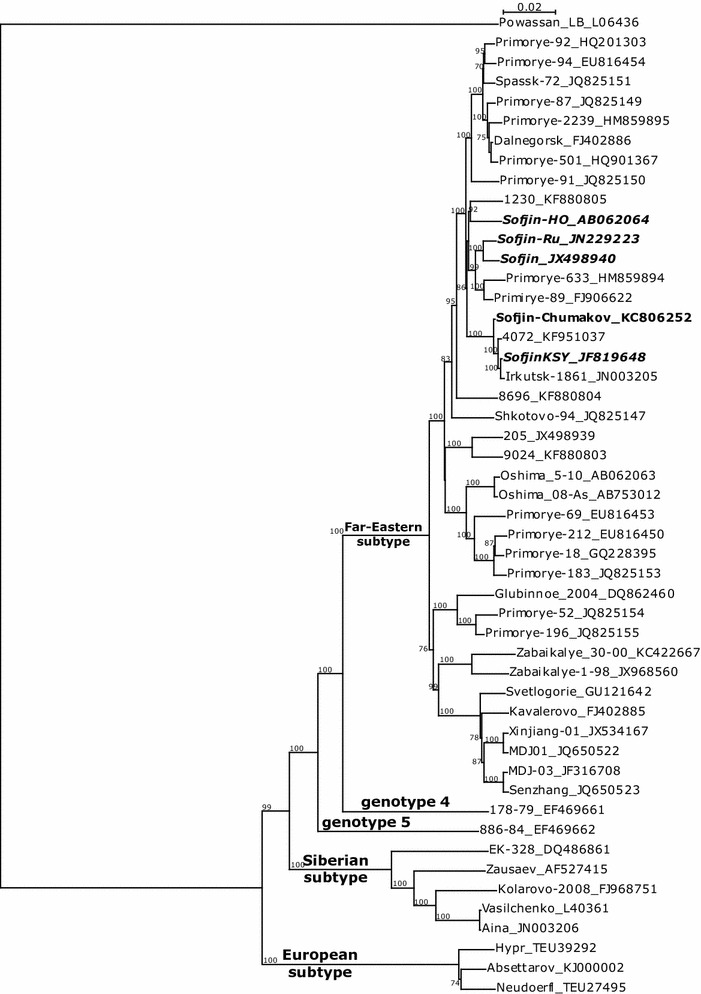
Phylogenetic tree of complete genome sequences of TBEV strains (NJ method). Strain Sofjin-Chumakov is shown in *bold*, other “Sofjin” strains are shown in *bold italics*

We performed a phylogenetic analysis with Clustal-X 2.0.11 (Larkin et al. 2007) of Sofjin-Chumakov and GenBank complete genome sequences of TBEV strains, including several “Sofjin” strains (Fig. 1). The Far-Eastern subtype showed several clusters. All “Sofjin” strains belonged to a group with several strains from the Urals, Primorskiy kray and Irkutsk region (Russia).

**Fig. 2:**
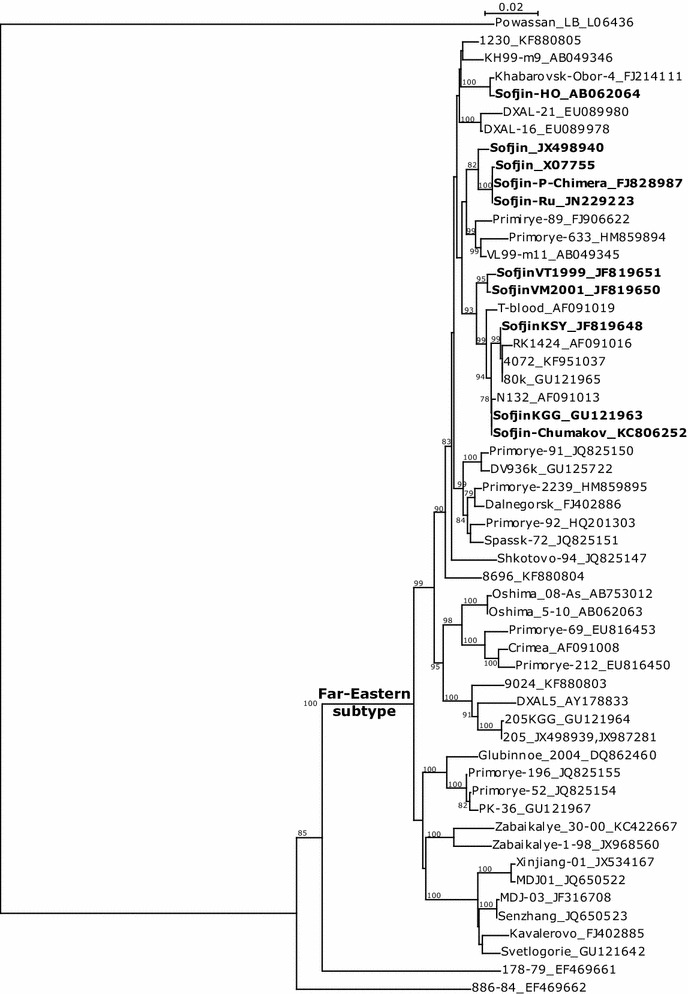
Phylogenetic tree of Far-Eastern TBEV subtype for fragment genome sequences for E protein, including all “Sofjin” strains (shown in *bold*) (NJ method)

The phylogenetic analysis of GenBank nucleotide sequences of protein E encoding genome fragments of Far-Eastern TBEV strains (Fig. 2) showed, as mentioned previously (Kovalev et al. 2012), that ‘Sofjin’ strains did not form a distinct branch on this tree. The E protein sequence of Sofjin-Chumakov was identical to the one of the TBEV strain SofjinKGG (Kozlovskaya et al. 2010). This strain has been used as the Sofjin strain in Chumakov IPVE for many years.

Strain SofjinKSY_JF819648 (D.I. Ivanovsky Institute of Virology) (Kovalev et al. 2012) contained only five nucleotide substitutions to the Sofjin-Chumakov sequence. Possibly, these differences could be the result of different passage histories of these viruses.

Sofjin-Chumakov showed the greatest number of differences with strain Sofjin-HO, sequenced by Japanese authors, which was more related to the Khabarovsk strains.

Sequences Sofjin_X07755 and Sofjin-P-Chimera_FJ828987 were almost identical to Sofjin-Ru_JN229223. Sofjin-P-Chimera was made using plasmid, prepared by Dobrikova and Pletnev (Dobrikova and Pletnev 1995) from the Sofjin strain, and sequenced by Pletnev’s team (Pletnev et al. 1986, 1990). Sofjin-Ru is a virus revived in vitro from the same plasmid. These three viruses, along with Sofjin_JX498940 (NSC ‘Vector’), formed a distinct cluster.

Sequence SofjinVM2001_JF819650 showed a significant difference from the sequence Sofjin-Chumakov, despite the fact that its authors claimed it to be sequenced from a drugstore-bought vial of TBE vaccine Moscow (Kovalev et al. 2012). It could be due to formalin inactivation damage to the viral RNA that finally caused various nucleotide mismatches during RT-PCR reactions before sequencing or any other laboratory artifacts.

We showed that the sequences of the virus on all stages of the vaccine manufacturing process are identical to the one which they all originate from, master seed bank.

The sequence of the working seed bank showed no nucleotide substitutions in passaged viruses from the sequence of the Sofjin-Chumakov from the master seed bank. Sequences of structural part of the non-concentrated bulk genomes were identical to the one from the master seed bank virus.

Sanger’s method of sequencing does not allow detecting up to 10 % of the admixture, genetically different from the master-sequence. The TBE vaccine Moscow is an inactivated vaccine, so the main role in the vaccine potency goes to inactivated virion antigen, which includes structural proteins only. More than 85–90 % identity of the structural proteins (antigen) throughout the manufacturing process is enough to imply the antigenic stability of the virus through the manufacturing process in the case of the inactivated vaccine.

## Conclusions

In the present work, we first described the genome of the TBEV strain Sofjin, which has the longest history as a vaccine strain, and then provided evidence of its stability during the manufacturing process.
